# Bridging the gap: a practical step-by-step approach to warrant safe implementation of large language models in healthcare

**DOI:** 10.3389/frai.2025.1504805

**Published:** 2025-01-27

**Authors:** Jessica D. Workum, Davy van de Sande, Diederik Gommers, Michel E. van Genderen

**Affiliations:** ^1^Department of Adult Intensive Care, Erasmus MC University Medical Center, Rotterdam, Netherlands; ^2^Department of Intensive Care, Elisabeth-TweeSteden Hospital, Tilburg, Netherlands; ^3^Erasmus MC Datahub, Erasmus MC University Medical Center, Rotterdam, Netherlands

**Keywords:** large language models, responsible AI, artificial intelligence, health care quality, access and evaluation, disruptive technology

## Abstract

Large Language Models (LLMs) offer considerable potential to enhance various aspects of healthcare, from aiding with administrative tasks to clinical decision support. However, despite the growing use of LLMs in healthcare, a critical gap persists in clear, actionable guidelines available to healthcare organizations and providers to ensure their responsible and safe implementation. In this paper, we propose a practical step-by-step approach to bridge this gap and support healthcare organizations and providers in warranting the responsible and safe implementation of LLMs into healthcare. The recommendations in this manuscript include protecting patient privacy, adapting models to healthcare-specific needs, adjusting hyperparameters appropriately, ensuring proper medical prompt engineering, distinguishing between clinical decision support (CDS) and non-CDS applications, systematically evaluating LLM outputs using a structured approach, and implementing a solid model governance structure. We furthermore propose the ACUTE mnemonic; a structured approach for assessing LLM responses based on Accuracy, Consistency, semantically Unaltered outputs, Traceability, and Ethical considerations. Together, these recommendations aim to provide healthcare organizations and providers with a clear pathway for the responsible and safe implementation of LLMs into clinical practice.

## Introduction

Large language models (LLMs) are artificial intelligence (AI) systems with the inherent capability of processing and interpreting natural language (Thirunavukarasu et al., 2023). LLMs show promise in transforming healthcare, offering a newfound flexibility in that, like a Swiss army knife, one single tool can be used for various applications, including administrative support and clinical decision-making (Schoonbeek et al., 2024; Levra et al., 2024). For example, LLMs can aid clinicians by efficiently summarizing medical records and crafting discharge documents. A recent study by Schoonbeek et al. demonstrated that the GPT-4 model proved to be as complete and correct as the clinician in summarizing clinical notes in preparation for outpatient visits, while being 28 times faster (Schoonbeek et al., 2024). Furthermore, LLMs have shown to offer a level of empathy in responding to patient questions that could surpass human clinicians (Ayers et al., 2023; Luo et al., 2024). Beyond these administrative or documentation tasks, the application of LLMs in healthcare can be expanded to clinical decision support. For example, when comparing the performance of an LLM to medical-journal readers in diagnosing complex real-world cases, the LLM outperformed its human counterparts with 57% vs. 36% correct diagnoses (Eriksen et al., 2023). These examples represent a mere subset of potential applications of LLMs in healthcare, with the scope continuously expanding at rapid pace.

**Figure 1:**
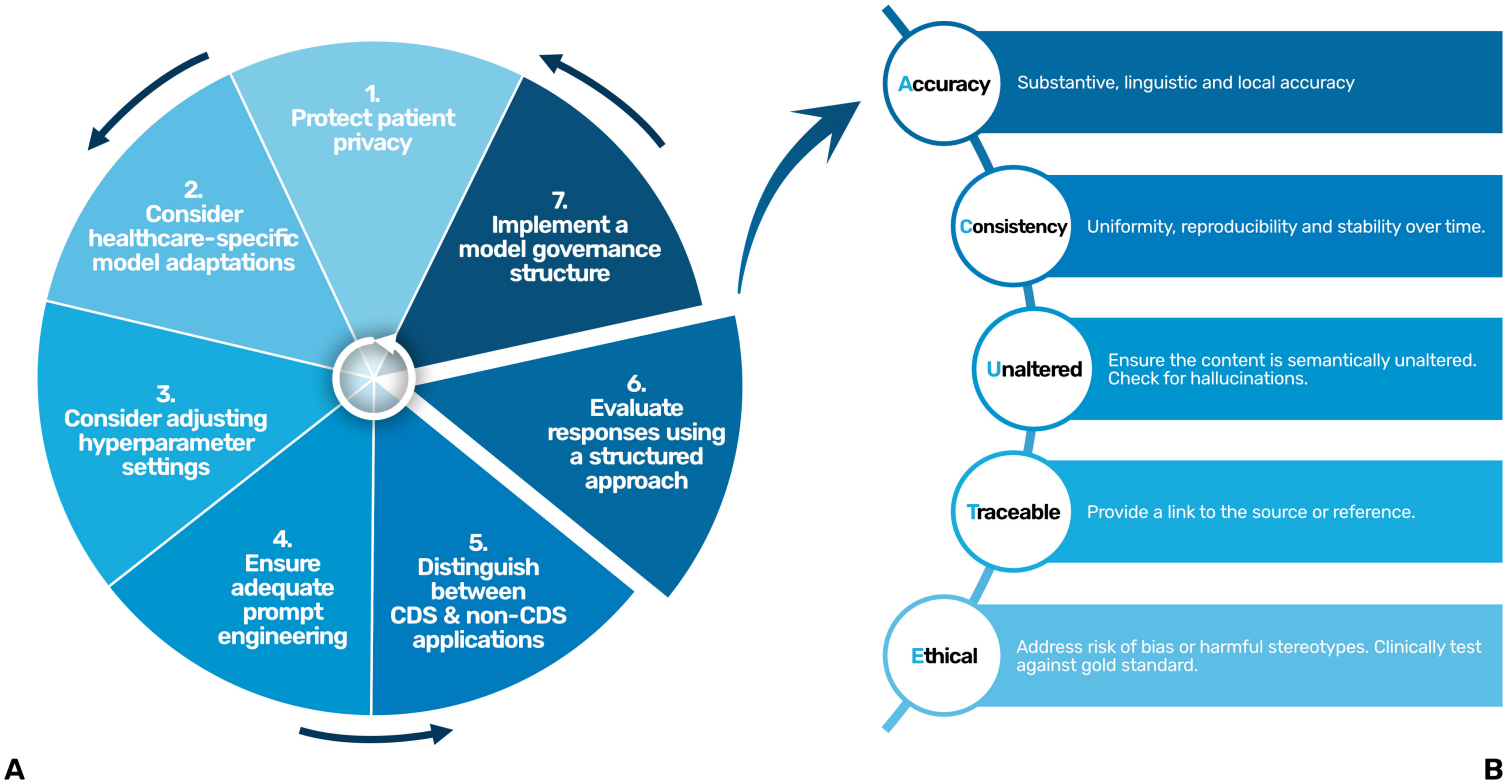
A step by step approach **(A)** and a structured evaluation approach (ACUTE) **(B)** for the responsible and safe use and development of LLMs in healthcare. CDS, Clinical Decision Support.

When used for clinical decision support (CDS), LLMs are likely to be considered a medical device and thus have to adhere to strict legislation, requiring thorough assessment to ensure quality standards (Keutzer and Simonsson, 2020; Jackups, 2023). However, for non-CDS applications, there is a lack of robust frameworks and regulatory oversight to ensure high quality output and responsible use of these models in clinical settings. Furthermore, existing legislations provide little guidance on responsible and safe implementation of LLMs from the healthcare organization or provider's perspective. This problem has also been identified recently the World Health Organization in their report on Ethics and Governance of AI for Health (World Health Organization, 2024). Current existing frameworks remain largely abstract and provide limited practical guidance (Raza et al., 2024). Thus, despite the growing use of LLMs in healthcare, a critical gap persists in clear, actionable guidelines for healthcare organizations and providers to ensure their responsible and safe implementation. In this paper, we propose a practical step-by-step approach, combined with an evaluation framework, to bridge this gap and support healthcare organizations and providers in warranting the responsible and safe implementation of LLMs into healthcare (Figure 1).

## (1) Protect patient privacy

LLMs have the potential to inadvertently reveal sensitive information to third parties if this information has been previously used as an input in the LLM (Open et al., 2023). Currently, protective measures (i.e., safeguards) to prevent such data leaks are inconsistent, leaving gaps in privacy protection (Yao et al., 2024). Importantly, in the context of healthcare, adherence to legal frameworks designed to safeguard personal data, such as the General Data Protection Regulation (GDPR) in Europe and the Health Insurance Portability and Accountability Act (HIPAA) in the U.S., is crucial. These regulations mandate that patient information must not be disclosed to third parties, including the developers or hosts of LLMs. Consequently, publicly available LLMs, which typically log user interactions for the purpose of model improvement (retraining), are not viable for healthcare applications due to the risk of data exposure and misuse. To improve user acceptance, LLMs should ideally be integrated into healthcare Information Technology (IT) ecosystems that host these models locally or on secure hospital-owned cloud servers (Nazari-Shirkouhi et al., 2023). This approach guarantees that patient data are securely maintained within the digital infrastructure of the hospital, thereby reinforcing the confidentiality and privacy of patient data. However, this is often not feasible due to high costs and infrastructure demands. Furthermore, the best performing general-purpose LLMs cannot be deployed locally due to proprietary nature of these models, restricting deployment on local servers. Open-source or smaller language models might be considered, but their performance can be inferior to proprietary LLMs (Wu et al., 2024).

It is paramount that if third party hosts of LLMs are used in healthcare, patient privacy is protected by stablishing a secure way of data transmission and guaranteeing that the data is not retained and the model is not retrained with user data. As such, an application programming interfaces (APIs) can serve as a secure connection between the hospital and the third party LLM host by implementing robust encryption protocols. Importantly, healthcare providers must be aware that they should establish strict contractual agreements with third-party LLM hosts to prevent data retention and ensure that user or patient data is not utilized for model retraining.

## (2) Consider healthcare-specific model adaptations

General-purpose LLMs still face performance limitations and may not suffice for complex and specialized healthcare tasks without modifications (Mao et al., 2023). Therefore, specific use cases might benefit from integrating medical domain knowledge in the language model. There are two main ways of doing so: by creating a healthcare specific language model or by adapting an existing LLM with medical domain knowledge, either through retraining or by giving it access to a database with specific medical knowledge.

Benefits of creating healthcare-specific models are that they could address challenges such as fairness, transparency, and data-inconsistency and might perform better for very specific medical domain knowledge (He et al., 2023). An additional benefit is that these models are typically smaller in size, leaving the possibility of running these models locally. However, it appears that the development of general-purpose LLMs is advancing more rapidly than that of healthcare-specific models, likely due to broader investment and scalability. By adapting an existing general-purpose LLM with medical domain knowledge, the performance of LLMs within the medical field increases dramatically (Ferber et al., 2024). This can be achieved either by periodically retraining the model with medical domain knowledge, or through Retrieval Augmented Generation (RAG), a technique that integrates an external knowledge database with an LLM through a pre-constructed index (Ng et al., 2025). Comparing both techniques to a human writer: with retraining, the memory of the writer has been expanded, and with RAG, the writer has continuous access to an up-to-date library of information. With RAG, the LLM is combined with a database of specific medical domain knowledge. The LLM draws information from this database when formulating a response, similar to a search engine. This ensures its responses are aligned with the latest medical knowledge while reducing the risk of hallucinations (Zakka et al., 2024). RAG significantly improves the performance of LLMs for healthcare-specific applications. For example, when connecting a RAG framework to international oncology guidelines, the LLM's response improved from 57% to 84% in answering questions correctly regarding the management of oncology patients (Ferber et al., 2024). Due to its flexibility, RAG is particularly beneficial in fields where knowledge evolves rapidly, such as medicine.

## (3) Consider adjusting hyperparameter settings

Another way of improving an LLM's output is by adjusting its hyperparameters, particularly its temperature setting. The temperature controls between the randomness of the generated responses. Higher temperatures generate more variability, while lower temperatures result in more predictable and consistent responses, adhering more closely to the provided prompts (Pugh et al., 2024). Therefore, it is thought that lower temperatures are recommended when consistency is important, whereas higher temperatures might be useful in addressing ambiguity. However, despite the rationale for adjusting temperature settings based on the specific demands of a clinical use case, recent available evidence suggests that adjustment of temperature has no significant effect on the consistency of performance for various LLMs across different clinical tasks, possibly rendering this step obsolete in the future (Patel et al., 2024).

## (4) Ensure adequate prompt engineering

An LLM's output is highly determined by the quality of the instructions or input to the model (prompt). Prompt engineering refers to the practice of designing and implementing prompts and is considered a new discipline within the field of AI. Advanced prompt engineering techniquesimprove the quality of the response of the model significantly (Zhang X. et al., 2024). Examples of advanced prompt engineering techniques are Few-Shot prompting and Chain-of-Thought (CoT) prompting. In Few-Shot prompting, the prompt includes a small number of examples to guide the model's understanding of the task. By providing these task-specific examples, the model is able to produce more accurate responses, even in scenarios where it has not been extensively trained. For example, in answering sample exam questions for the United States Medical Licensing Examination, 5-shot prompting improved the performance for the GPT-4 model from 84% to 87% correct (Nori et al., 2023). In CoT prompting, the model is instructed to engage in step-by-step reasoning by breaking down complex questions into smaller steps (Wei et al., 2022). This structured approach helps the model reason through tasks more effectively, improving coherence and accuracy of the outputs. CoT is especially useful for tasks requiring logical progression, making this technique of particular interest in CDS applications (Miao et al., 2024). However, various other prompt optimization approaches exist, reflecting the rapid evolution of this new discipline (Chang et al., 2024).

Currently, healthcare professionals rely heavily on extensive experimentation using LLMs, with a limited theoretical understanding of why a specific phrasing or formulation of a task is more sensible than others. Inadequate prompt engineering in medicine without strict constraints could lead to undesired outputs, such as (erroneous) medical advice. It is therefore vital that prompts in the medical field should be created by experts in medical prompt engineering (Chen et al., 2024).

## (5) Distinguish between CDS and non-CDS applications

Due to regulatory oversight that warrant safe use of innovations in healthcare such as the Medical Device Regulation (MDR) in the European Union (EU) and the Food and Drug Administration (FDA) in the United States, it is important to differentiate between Clinical Decision Support (CDS) and non-CDS for the specific applications of LLMs. This differentiation strongly indicates whether the application is considered to be a medical device, and thus would fall under these specific regulations. CDS is generally understood to be any tool that assists clinicians in diagnostics or treatment decisions, and when it is used to inform clinical decisions that directly impact patient care, it is considered a medical device and would fall under these laws. In contrast, software that only provides supplementary information without driving clinical decisions, is not considered clinical decision support (non-CDS) and thus may not be classified as a medical device.

Consequently, an LLM that supports diagnostic or treatment processes would be classified as a medical device under, for example, the MDR. This prohibits the use of the tool until it has undergone a thorough assessment to ensure that it meets MDR-related quality standards, such as providing clinical evidence of their safety and effectiveness. This process may be time-consuming, possibly limiting the adoption of LLMs for CDS in healthcare.

Unlike traditional medical devices or AI-models, LLMs are inherently multi-purpose, capable of addressing diverse clinical and non-clinical queries. Subjecting LLMs to regulatory approval for each specific clinical purpose is impractical due to the immense effort and cost required. Their rapid evolution, with frequent updates in data, methods, and architectures, further complicates regulation. Regulatory sandboxes offer a supervised setting to explore regulatory requirements and evaluate LLM performance iteratively, providing a flexible pathway to address these challenges.

### CDS applications

The use of LLMs for CDS seems very promising. When presented with United States Medical Licensing Examination (USMLE) sample exam questions, the GPT-4 model correctly answered 87% without any healthcare-specific adaptations (Nori et al., 2023). Additionally, on various publicly available benchmark datasets, such as the MedQA and the medical components of the Massive Multitask Language Understanding (MMLU), the GPT-4 model performed outstandingly well, answering over 80% correct for each benchmark (Nori et al., 2023). This indicates that general medical knowledge is inherently present in these models. Fewer studies have researched the capabilities of LLMs for specialized medical knowledge within clinical subdomains. For example, in a recent study, the GPT-4 model was able to correctly answer nephrology questions with a score of 73%, without healthcare-specific adaptations or advanced prompt engineering techniques, indicating its potential for highly specialized fields (Wu et al., 2024). When compared to human physicians, the performance of the GPT-4 model exhibited variation across medical specialties, although the model consistently met or exceeded the examination threshold in the majority of cases (Katz et al., 2024).

When implemented into healthcare, CDS will most likely require the use of healthcare-specific model adaptations, utilizing techniques such as RAG, to improve the accuracy of responses. Relevant references should be linked so that the source of the information can be checked.

However, the progression toward CDS necessitates more than the mere capability to answer clinical questions, as clinical decision-making encompasses a combination of medical knowledge, clinical reasoning, multidisciplinary collaboration, evidence-based practice and communication skills. Current advancements in LLMs, aimed at improving logical reasoning, bring the use of LLMs for CDS closer to fruition. Nevertheless, due to the potential significant impact on clinical decision-making, implementing LLMs for CDS demands tremendous diligence.

### Non-CDS applications

The majority of non-CDS applications aims to reduce the administrative load for healthcare providers. Various examples are currently being implemented, such as composing draft responses to patient messages and creating summaries of the patient chart (Schoonbeek et al., 2024; van Veen et al., 2023; Garcia et al., 2024; Tai-Seale et al., 2024). If a use case is not considered CDS, there are currently no laws or guidelines in place to ensure responsible and safe use of LLMs. Given the swift development and adoption of LLMs in society, it is likely that additional non-CDS applications of LLMs are coming to healthcare rapidly.

While legal frameworks such as the EU AI Act, GDPR, and HIPAA establish important baseline requirements for data protection and accountability, they do not address the unique challenges posed by LLMs in clinical settings. For example, they lack requirements for clinical validation, i.e., objectively assessing whether outputs are sufficient for clinical use while accounting for risks like hallucinations, missing information and misinterpretations. These challenges underscore the need for healthcare-specific validation processes to complement existing legal frameworks.

## (6) Evaluate using a structured approach

To ensure the responses of the LLM remain accurate, consistent, and aligned with clinical standards over time, a structured approach to evaluate their responses is essential. As LLMs are probabilistic by nature, their performance can vary, making continuous and systematic evaluation critical for maintaining quality and preventing errors, especially in high-stakes environments such as healthcare. Abbasian et al. proposed an extensive set of intrinsic and extrinsic evaluation metrics for assessing the performance of healthcare chatbots, including evaluating the quality of their response (Abbasian et al., 2024). However, their comprehensiveness limits their practicality in clinical settings. To balance comprehensiveness and simplicity, we've identified five key points that should be addressed when evaluating the response of an LLM in clinical settings, being accuracy, consistency, semantically unaltered, traceable and ethical. The mnemonic “ACUTE” (Figure 1B) could be used as a helpful tool.

Accuracy encompasses three domains: first, substantive accuracy, meaning that responses are factually correct and contextually appropriate within the medical field, even for non-clinical decision support (non-CDS) applications. When determining if a response is substantively accurate, it is important to determine if the response is complete (i.e., determine if there is any information missing) and correct (i.e., determine if there are any factual errors). The second domain is linguistic accuracy, particularly for languages other than English. As foundational models are predominantly trained on English data, responses may exhibit reduced accuracy in other languages. Rigorously test for linguistic accuracy by adjusting the prompt. Frequently, writing the prompt in English and asking the LLM to translates yields better results. The third domain is local accuracy, which means, ensuring that the responses reflect each hospital's own policies and communication preferences.

When deployed in clinical practice, LLM responses need to be reproducible and stable over time, ensuring reliability in their outputs. As such, consistency is another key criterion. If the LLM provides inconsistent results, try adjusting the temperature settings or the prompt. If the inconsistency remains, try a different LLM for this specific clinical task.

The responses should also be semantically Unaltered. The response of LLM should accurately reflect the information presented in the patient chart without introducing extraneous content (hallucinations). Furthermore, the responses should be Traceable, making it clear where the LLM obtained its information, ideally by providing a reference to the source. For example, when utilizing RAG, the source of the information should be cited, and when summarizing notes in the patient chart, after each claim, the original note should be linked.

And lastly, the Ethical dimension mandates the responsible use of LLMs and aims to prevent that LLM responses do not perpetuate biases or harmful stereotypes, ensuring the responsible and fair use of these models in clinical practice. LLMs are typically trained on large datasets that include publicly available text, which often contains inherent biases reflective of societal inequalities. Studies have shown that these biases can perpetuate in LLM outputs, leading to disparities in diagnosis and treatment across different demographic groups. The Benchmark of Clinical Bias in Large Language Models (CLIMB benchmark) highlights how LLMs may exhibit these biases, resulting in unequal diagnostic accuracy across populations (Zhang Y. et al., 2024). Similarly, another study found that LLMs could reinforce harmful stereotypes, such as underdiagnosing conditions like smoking in young males and obesity in middle-aged females (Pal et al., 2023). This emphasizes the need for careful oversight to prevent biased decision-making in clinical practice. Ideally, each new use case should be clinically tested compared to its gold standard, which is generally the performance of the clinician.

In contrast to existing frameworks that provide broad, cross-sectoral guidelines, the ACUTE framework offers a specialized and practical approach tailored to the unique requirements of healthcare, focusing specifically on evaluating LLM outputs for clinical relevance and patient safety. We believe that using the ACUTE mnemonic as a structured approach balances simplicity and comprehensiveness for the evaluation of LLM responses and remains practical for real-world clinical use while still adequately addressing key challenges in LLM evaluation and deployment. Comparative analyses utilizing the ACUTE framework should be performed between LLM-generated outputs and clinician outputs for clinical validation.

## (7) Implement a model governance structure

Eventually, it is crucial to ensure high quality performance and output of the LLM over time and therefore, a system for regular monitoring and continuous evaluation should be in place. This is of particular importance, as an LLM's performance can vary over time via retraining or is updated to a new version. Thus, establishing a governance framework to monitor the LLM's performance over time and implement adaptive maintenance strategies is crucial. In addition to model governance, robust data governance is essential, ensuring transparent data management and controlled access. Governance principles for both data and models should be traceable, securely stored, and readily accessible to notified bodies and competent authorities to support regulatory compliance. A dedicated team comprising medical and AI experts should be established to collect and evaluate user feedback, interpret model quality and outputs, and implement appropriate actions accordingly. Adaptive maintenance strategies could include periodic audits of LLM outputs and robust fallback mechanisms, such as maintaining access to legacy versions and options for model switching. By incorporating these measures, the governance structure will remain robust and futureproof, safeguarding both safety and reliability over time. The ACUTE framework mentioned in step 6 could offer such guidance.

## Connect all the steps

To move toward the safe and responsible development and implementation of LLMs in both administrative tasks and clinical decision support in healthcare, connecting all the steps is essential. For example, by combining different prompt engineering techniques with healthcare-specific model adaptations like RAG the overall performance of an LLM on medical board examinations improves significantly, highlighting the importance of considering the steps outlined in this manuscript (Samaan et al., 2024). As a practical aid, we have transformed the recommendations into “critical questions” in Table 1, and the ACUTE framework into a checklist in Table 2. These critical questions are designed to assess the readiness for responsible LLM implementation in healthcare. If these questions cannot be answered adequately, there is a significant gap that must be addressed prior to utilizing LLMs in healthcare. The ACUTE checklist will help systematically evaluate the performance of an LLM application, while highlighting potential weaknesses.

**Table 1:** Critical questions to guide responsible LLM implementation in healthcare with actionable steps.

**Recommendation**	**Critical questions**
1. Protect patient privacy	How is patient data securely transmitted and stored?
	Are third-party agreements in place to prevent data retention or model retraining?
	Are the LLMs hosted on secure, hospital-controlled infrastructure?
2. Consider healthcare-specific model adaptations	Is medical domain knowledge paramount to the specific use case for which an LLM is deployed?
	Has the LLM been adapted or validated for the specific healthcare tasks it will perform? If so, how?
	Does the application utilize RAG (Retrieval-Augmented Generation) to integrate up-to-date medical knowledge?
3. Consider adjusting hyperparameter settings	Have hyperparameters, such as temperature, been adjusted to align with the specific clinical use case?
	Has the impact of hyperparameter adjustments been adequately evaluated?
4. Ensure adequate prompt engineering	Who is responsible for writing and maintaining the prompts?
	Have medical professionals been involved in designing and testing the prompts?
	Have the prompts been tested and refined in an iterative manner to minimize errors and undesired outputs?
5. Distinguish between CDS and non-CDS applications	Is the application clearly categorized as either Clinical Decision Support (CDS) or non-CDS?
	For CDS applications, does the LLM comply with potentially relevant medical device regulations (e.g., MDR, FDA)?
	For non-CDS applications, are barriers set in place to avoid unintended use as a medical device?
6. Evaluate using a structured approach	Are LLM outputs evaluated using a structured framework, such as the ACUTE criteria (Accuracy, Consistency, Unaltered meaning, Traceability, Ethical considerations)?
	Is there a process for documenting evaluation results and using them to guide improvements?
7. Implement a model governance structure	Is there a dedicated team in place to monitor and oversee LLM performance over time?
	Are evaluations performed regularly to ensure ongoing alignment with clinical standards?
	Are fallback mechanisms established to ensure continuity?

**Table 2:** Checklist for the ACUTE framework, designed to evaluate LLM outputs in healthcare and ensure that each criterion is addressed effectively to minimize risks and enhance reliability.

**Dimension**	**Criteria**	**Focus**
Accuracy	Are responses factually correct and complete?	Substantive accuracy
	Are responses grammatically correct and clear, even in non-English languages?	Linguistic accuracy
	Do responses align with hospital policies and preferences?	Local accuracy
Consistency	Are responses consistent across repeated prompts?	Reproducibility
	Are responses stable across different sessions and model versions?	Stability over time
	Are inconsistencies addressed effectively through prompt refinements?	Mitigation of inconsistencies
Unaltered	Do responses avoid adding erroneous or fabricated information?	Hallucinations
	Do responses accurately reflect the input data, such as patient charts?	Reflection of source data
Traceability	If applicable, are claims and recommendations clearly linked to credible sources?	Source attribution
	If applicable, are external references provided when RAG or other systems are used?	Use of retrieval systems
Ethical	Do responses avoid perpetuating harmful biases or stereotypes?	Bias avoidance
	Are sensitive topics handled responsibly and respectfully?	Sensitive topics

Ultimately, we must bridge the gap between technological AI model development and trustworthy and responsible AI adoption in a clinical setting. Despite the growing use of LLMs, a critical gap persists in clear, actionable guidelines available to healthcare organizations and providers to ensure their responsible and safe implementation. The integration of a step-by-step approach, combined with a practical evaluation framework, could address this gap. By balancing simplicity with comprehensiveness, these recommendations could lower AI hesitancy, improve clinical implementation and unlock its full potential in improving healthcare. Future researchers are encouraged to validate the proposed framework across diverse clinical scenarios. Advancing the responsible implementation of LLMs in healthcare will require a collective effort from healthcare organizations, providers, researchers, and policymakers to ensure robust validation, responsible use and adequate monitoring of LLMs in clinical practice. The recommendations outlined in this manuscript provide a practical starting point for this collaborative journey, offering guidance for the responsible and effective implementation of LLMs in healthcare.

## Data Availability

The original contributions presented in the study are included in the article/supplementary material, further inquiries can be directed to the corresponding author.
